# The Role of Artificial Intelligence in the Diagnosis and Management of Diabetic Retinopathy

**DOI:** 10.3390/jcm14145150

**Published:** 2025-07-20

**Authors:** Areeb Ansari, Nabiha Ansari, Usman Khalid, Daniel Markov, Kristian Bechev, Vladimir Aleksiev, Galabin Markov, Elena Poryazova

**Affiliations:** 1 Faculty of Medicine, Medical University of Plovdiv, 4002 Plovdiv, Bulgaria; nabiha.m.ansari@gmail.com (N.A.); usmankhalid957@gmail.com (U.K.); gabi_markov@abv.bg (G.M.); 2 Department of General and Clinical Pathology, Medical University of Plovdiv, 4002 Plovdiv, Bulgaria; daniel.markov@mu-plovdiv.bg (D.M.); kristian_bechev@abv.bg (K.B.); eporiazova@abv.bg (E.P.); 3 Department of Clinical Pathology, University Multidisciplinary Hospital for Active Treatment “Pulmed”, 4002 Plovdiv, Bulgaria; 4 Neurological Surgery, Pulmed University Hospital, 4000 Plovdiv, Bulgaria; 5 Department of Thoracic Surgery, University Hospital “Kaspela”, 4002 Plovdiv, Bulgaria; vl_alex@abv.bg; 6 Department of Cardiovascular Surgery, Medical University of Plovdiv, 4002 Plovdiv, Bulgaria

**Keywords:** diabetic retinopathy, diabetes mellitus, artificial intelligence, screening, healthcare accessibility

## Abstract

**Background/Objectives**: Diabetic retinopathy (DR) is a progressive microvascular complication of diabetes mellitus and a leading cause of vision impairment worldwide. Early detection and timely management are critical in preventing vision loss, yet current screening programs face challenges, including limited specialist availability and variability in diagnoses, particularly in underserved areas. This literature review explores the evolving role of artificial intelligence (AI) in enhancing the diagnosis, screening, and management of diabetic retinopathy. It examines AI’s potential to improve diagnostic accuracy, accessibility, and patient outcomes through advanced machine-learning and deep-learning algorithms. **Methods**: We conducted a non-systematic review of the published literature to explore advancements in the diagnostics of diabetic retinopathy. Relevant articles were identified by searching the PubMed and Google Scholar databases. Studies focusing on the application of artificial intelligence in screening, diagnosis, and improving healthcare accessibility for diabetic retinopathy were included. Key information was extracted and synthesized to provide an overview of recent progress and clinical implications. **Conclusions**: Artificial intelligence holds transformative potential in diabetic retinopathy care by enabling earlier detection, improving screening coverage, and supporting individualized disease management. Continued research and ethical deployment will be essential to maximize AI’s benefits and address challenges in real-world applications, ultimately improving global vision health outcomes.

## 1. Introduction

Diabetic retinopathy (DR) is a chronic, progressive complication of diabetes mellitus (DM) and remains a leading cause of vision impairment and blindness among the working-age population worldwide [1,2]. As a microangiopathy, DR arises from long-standing metabolic dysregulation and is now understood to be a complex inflammatory and neurovascular disorder, in which neuronal injury often precedes clinically detectable microvascular damage [1,3]. The disease typically progresses through well-defined stages, from non-proliferative diabetic retinopathy (NPDR) to proliferative diabetic retinopathy (PDR), with the latter carrying significant risk of irreversible vision loss and being predictive of other severe systemic complications such as stroke and cardiovascular events [1,2,4]. Despite the implementation of guidelines recommending annual screening for all individuals with diabetes, real-world detection and timely intervention remain suboptimal [2,5]. The traditional model of DR diagnosis is reliant on fundus photography and ophthalmologist interpretation, which faces several challenges. These include a shortage of eye care professionals, variability in diagnostic grading, and the need for high-resource infrastructure, particularly in underserved regions [5,6]. Moreover, a substantial proportion of patients with diabetes remains undiagnosed or presents only after irreversible retinal damage has occurred [2,3]. This underscores the urgent need for accessible, scalable, and cost-effective screening and management tools that can identify DR at its earliest stages and mitigate progression to vision-threatening disease.

In this context, artificial intelligence (AI) has emerged as a transformative tool with the potential to reshape DR care. AI, particularly through deep-learning algorithms, has demonstrated high sensitivity and specificity in detecting referable DR from retinal images, rivaling expert human graders [6,7]. These AI systems are now capable of autonomous or semi-autonomous diagnoses, DR stage classification, and even real-time disease progression monitoring [4,7]. Additionally, AI technologies offer promise in extending eye care access via telemedicine, particularly in low-resource settings where the burden of diabetes is rapidly increasing [4].

Recent efforts have focused not only on improving diagnostic accuracy but also on addressing practical challenges in deploying AI solutions into clinical practice. These include algorithm validation across diverse populations, integration into electronic health records, and navigation of ethical concerns such as data privacy and algorithmic bias [5,6]. As AI tools evolve from development to real-world deployment, there is growing interest in their role beyond screening in prognostication, treatment planning, and individualized disease monitoring [5,7].

This literature review explores the multifaceted role of AI in the diagnosis and management of diabetic retinopathy. It examines the pathophysiology of DR, such as microvascular damage, capillary leakage, and retinal ischemia, which leads to subtle retinal changes before clinical symptoms appear. These early alterations provide a foundation for AI-based systems to detect disease at a subclinical stage using high-resolution retinal imaging. In addition, this article reviews the current landscape of AI technologies and their clinical validation and highlights implementation challenges and opportunities for improving global DR care through predictive, preventive, and personalized medicine.

## 2. Materials and Methods

This review was conducted as a narrative literature review aimed at evaluating current evidence on the application of AI in the diagnosis and management of DR. A comprehensive search was performed using PubMed, Scopus, and Google Scholar for articles published up to May 2025. Only English-language articles were included. The search terms used were: “artificial intelligence,” “machine learning,” “deep learning,” “diabetic retinopathy,” “screening,” and “diagnosis.”

Studies were eligible for inclusion if they (1) discussed AI in relation to DR and (2) were full-text original articles or review papers containing essential sections (introduction, methods, results, and discussion). Data were extracted and synthesized focusing on study design, population, AI models used, performance metrics (e.g., sensitivity, specificity, and area under the curve (AUC)), and practical outcomes such as accessibility and follow-up adherence.

Although a variety of study types were included (e.g., randomized controlled trials, retrospective studies, and real-world implementations), we qualitatively addressed the heterogeneity by categorizing findings based on study design and highlighting methodological differences in the discussion. This approach allowed for the comparison of evidence while acknowledging variations in study type, setting, and applicability.

**Figure 1:**
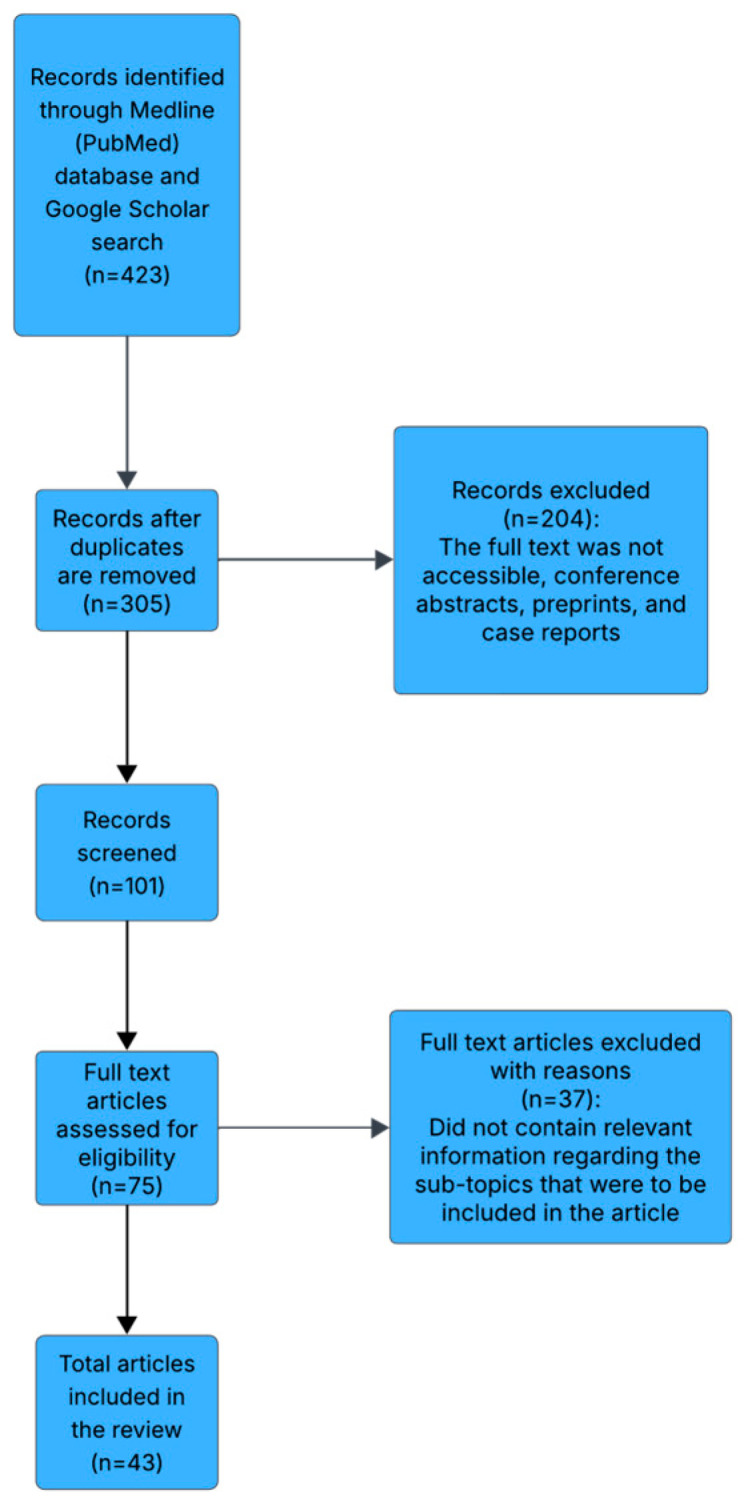
Methods of selecting the final articles included in this review.

Initial screening of titles and abstracts was followed by a detailed review of full-text articles. The search was supplemented by examining reference lists of selected papers to identify additional relevant studies. Final article selection was achieved through consensus among the authors to ensure relevance and inclusion quality (Figure 1).

## 3. Results

### 3.1. AI in Screening Programs and Accessibility

AI has emerged as a transformative tool in the screening of diabetic retinopathy, addressing critical barriers such as limited access to specialist care, delayed diagnosis, and poor follow-up adherence. AI algorithms, particularly those leveraging deep learning, are increasingly being implemented in both high-income and resource-limited settings, where they have demonstrated strong potential in improving screening uptake, diagnostic accuracy, healthcare efficiency, and patient outcomes.

The RAIDERS randomized controlled trial investigated whether AI-supported DR screening could improve referral uptake among patients with diabetes in Rwanda. Of the 827 patients screened, 275 (33.4%) met referral criteria based on AI interpretation and were randomized to receive either immediate AI-generated feedback or delayed referral advice after human grading. Referral adherence was significantly higher in the AI intervention group (51.5%) compared to the control group (39.6%), with a 30.1% increase in adherence (*p* = 0.048). Multivariate analysis confirmed that being in the AI group, along with factors such as older age, male sex, and rural residence, was significantly associated with higher referral uptake. Additionally, patients in the AI group presented for referral care significantly sooner than those in the control group [8].

An RCT conducted in Northern Tanzania evaluated whether AI-supported DR screening can increase follow-up attendance among patients with true referable DR. The study addresses critical gaps in current DR screening in low-resource settings, such as limited specialist staff, delays in result delivery, and poor follow-up rates. Participants with diabetes (*n* = 2364) are randomized to either a standard care pathway, where images are graded later by clinicians with results delivered via phone, or an AI-supported pathway that provides immediate automated grading and face-to-face counselling at the time of screening. The primary outcome is the proportion of patients with a true referable DR who attend the central ophthalmology clinic within 8 weeks. Secondary outcomes include the sensitivity and specificity of AI versus standard grading, false positive rates, and an economic evaluation. The AI software used (SELENA + AI version 2.0) was selected for its regulatory approval, offline capability, and validated performance in African populations. In addition to demonstrating clinical benefits, this study incorporated a cost-effectiveness analysis, highlighting AI’s potential to reduce the economic burden of blindness and streamline health expenditures in sub-Saharan Africa [9].

In high-resource environments, autonomous AI systems have also improved access and follow-up care. The ACCESS trial, a U.S.-based study involving youth (ages 8–21) with diabetes, demonstrated the utility of autonomous AI in increasing screening rates in a primary care setting. The study found that exam completion was significantly higher in the AI group (100%) compared to the control group receiving standard referral and education (22%) (*p* < 0.001). Furthermore, 64% of those in the AI group who were flagged for diabetic eye disease followed up with an eye care provider, compared to just 22% in the control group (*p* < 0.001). While disparities in prior eye exams were linked to race, ethnicity, and socioeconomic status in univariate analysis, multivariate analysis revealed that the duration of diabetes was the only significant predictor [10].

One study evaluated the performance of an automated deep-learning-based retina image analysis (ARIA) system in classifying retinal fundus images as referable or non-referable DR cases in both international and Mexican populations. The ARIA system demonstrated high diagnostic accuracy, achieving area under the receiver operating characteristic curve (AUROC) values of 98% and 98.3%, sensitivities of 95.1% and 95.2%, and specificities of 91.5% and 90% for international and Mexican cases, outperforming the average performance of 17 ophthalmologists. When used as an assistive tool, ophthalmologists’ sensitivity significantly improved from 87.3% to 93.3% (*p* = 0.05), and specificity also increased. However, additional visual aids, such as attention maps and confidence levels, yielded mixed results with improved sensitivity but reduced specificity and greater performance variability [11].

Dow et al. in their study evaluated follow-up rates after DR screening across three workflows: a fully AI-based system, a traditional human-based system, and a hybrid AI–human system. Among 2243 screened patients, 279 tested positive for more-than-mild DR (MTMDR) via the AI workflow, with 69.2% following up with an ophthalmologist within 90 days, a follow-up rate roughly three times higher than the 12% and 11.7% seen in the human and hybrid workflows, respectively. The AI workflow’s faster result turnaround (within 48 h) likely contributed to improved adherence. Most patients who followed up did so based on primary care physician referrals. Barriers to follow-up among nonadherent patients included lack of knowledge about the need for further care, language issues, scheduling difficulties, and fear of diagnosis. Demographic and socioeconomic factors did not significantly influence follow-up behavior [12].

A backpropagation neural network was evaluated for its ability to detect key features of DR vessels, exudates, and haemorrhages in fundus photographs, with its diagnostic performance compared to that of an ophthalmologist. The network demonstrated high detection rates for vessels (91.7%) and exudates (93.1%), and a moderate rate for haemorrhages (73.8%). In the primary evaluation using 200 diabetic and 101 normal images, the neural network achieved a sensitivity of 88.4% and specificity of 83.5% for DR detection when compared to the ophthalmologist’s assessment. Adjusting the detection threshold could further improve sensitivity (up to 99%) at the expense of specificity [13].

Large-scale prospective studies have validated the real-world applicability of AI. In China, a national deep-learning system was evaluated across 47,000 patients at multiple sites. The deep-learning (DL) system demonstrated strong performance, with a sensitivity of 83.3% and specificity of 92.5% for detecting referable DR, and a grading concordance (83.0%) comparable to interobserver variability among specialists. Of the 40,665 gradable participants, the DL algorithm identified a DR prevalence of 28.8%, referable DR at 24.4%, and vision-threatening DR at 10.8%. DR prevalence was higher in females, older individuals, and those with longer diabetes duration or elevated HbA1c levels [14].

The EyeArt v2.1 AI algorithm was used in triaging retinal images from the English Diabetic Eye Screening Programme (DESP) into test-positive or test-negative categories, using manual human grading as the reference. Among 30,405 screening episodes from three centres, EyeArt demonstrated a high sensitivity of 95.7% for detecting referable DR, including 100% sensitivity for both moderate-to-severe non-proliferative and proliferative disease. Sensitivities for specific subgroups, such as R1M1, were also high (98.3%). However, specificity was lower (68%) for patients with no retinopathy (R0M0) and 54% when combined with non-referable cases (R1M0), indicating a relatively high false-positive rate [15].

A multicenter study by the U.S. Veterans Affairs system evaluated seven AI algorithms using over 311,000 fundus images from nearly 24,000 veterans. The performance of these screening algorithms was compared to human graders to assess their real-world effectiveness in detecting referable DR. Sensitivities of the algorithms varied significantly (50.98–85.90%), with only three (algorithms E, F, and G) showing comparable or superior sensitivity to human graders for moderate NPDR or worse. One algorithm (G) achieved similar sensitivity (80.47%) and specificity (81.28%) to VA teleretinal graders based on an arbitrated reference set, while others either lacked sensitivity or specificity. Notably, some algorithms failed to match human performance in detecting proliferative DR. Although negative predictive values were high (82.72–93.69%), especially in the Atlanta subset, positive predictive values were generally modest, emphasizing the need for extensive external validation and performance benchmarking before clinical deployment of AI systems [16].

In a related study, J. He et al. evaluated the accuracy and feasibility of using an AI-based system (Airdoc) for DR screening in a community hospital setting, involving 889 diabetic patients and 3556 retinal images. Compared to ophthalmologist grading, the AI system demonstrated high diagnostic performance, detecting any DR with 90.79% sensitivity and 98.5% specificity (AUC 0.946), and referable diabetic retinopathy (RDR) with 91.18% sensitivity and 98.79% specificity (AUC 0.950). The AI and ophthalmologists identified DR in a similar proportion of participants (16.3% vs. 16.1%) and RDR in 11.6% vs. 11.4%, respectively [17].

A real-world study in China assessed the performance of an AI-based DR grading model in a community clinic setting. Among 193 eligible diabetic participants, 173 (89.6%) had at least one readable eye image. AI and a panel of retina specialists evaluated 321 eyes, with a substantial agreement (κ = 0.715) in DR grading. For detecting RDR, the AI system demonstrated a sensitivity of 84.6% and specificity of 98.0% (AUC 0.913), while for any DR, it showed 90.0% sensitivity and 96.6% specificity (AUC 0.933), indicating that the AI model had good consistency with expert grading, with high specificity and acceptable sensitivity for DR and RDR detection [18].

A study in Zambia evaluated the accuracy of a deep-learning-based AI system for detecting DR in a population-based screening program. Using an ensemble model trained on Singaporean data, the AI system was tested on 4504 fundus images from 1574 Zambian patients. The AI demonstrated high diagnostic performance, with an AUC of 0.973, sensitivity of 92.25%, and specificity of 89.04% for referable DR. It also showed excellent sensitivity for detecting vision-threatening DR (99.42%) and diabetic macular oedema (97.19%). The AI model performed comparably to retinal specialists in both disease detection and identification of systemic risk factors (longer diabetes duration, elevated HbA1c, and higher systolic blood pressure) [19].

In a real-world validation study, the IDx-DR 2.0 device was validated in a real-world setting using retinal images from patients with type 2 diabetes in the Hoorn Diabetes Care System. Compared to grading by three retinal specialists using European diabetes study (EURODIAB) and international clinical diabetic retinopathy (ICDR) criteria, IDx-DR showed strong diagnostic performance for detecting RDR, with a sensitivity of 91% and specificity of 84% under EURODIAB, as well as a negative predictive value (NPV) of 100%. Under ICDR, sensitivity was 68%, specificity was 86%, and NPV was 97%. For vision-threatening DR (VTDR), sensitivity was lower (~62–64%), but specificity exceeded 95%. The AUC values ranged from 0.87 to 0.94, supporting IDx-DR’s use in primary care to reduce unnecessary specialist referrals [20].

A recent study in Spain demonstrated the effectiveness of a custom AI tool, NaIA-RD, in enhancing diabetic retinopathy (DR) screening. Integrated into an existing GP-led workflow, NaIA-RD combined DR detection and image quality assessment and was evaluated in a large before-and-after study involving over 42,000 patients. The AI significantly improved the sensitivity of general practitioners while maintaining high specificity, and its screening proposals showed strong agreement with ophthalmologists for referable cases (Cohen’s kappa = 0.818). When used autonomously, NaIA-RD reduced clinician workload by over four times without missing any cases of sight-threatening DR. It outperformed general practitioners in both DR severity grading and image quality classification, achieving a referable DR sensitivity of 96.9% vs. 87.1% for GPs, and matched or exceeded ophthalmologist performance across tasks, including on external benchmarks like Messidor-2 and APTOS 2019 [21] (Table 1).

### 3.2. Risk Prediction and Progression Modeling

Recent advancements in AI have enabled the development of deep-learning (DL) and automated machine-learning (autoML) models capable of accurately predicting the progression of DR. These models leverage retinal imaging data to detect subtle changes that precede clinical progression, offering new avenues for earlier intervention and personalized care.

In a retrospective study utilizing the EyePACS dataset, researchers aimed to predict the progression of DR from non-referable (NRDR) to referable (RDR) using AI models(ResNeXt-50) trained on longitudinal fundus images. A total of 12,768 images from 6384 eyes (two per eye, taken approximately 2 years apart) were analyzed. The ResNeXt DL model, trained at varying image resolutions, achieved high performance with AUC values up to 0.971 for detecting RDR [22].

Another study explored the utility of autoML models to predict DR progression using ultra-widefield (UWF) retinal images from 1179 patients with mild or moderate NPDR and 3 years of follow-up. The models performed particularly well in moderate NPDR cases, with AUPRC values of 0.717 for mild and 0.863 for moderate NPDR. In the validation set, they identified progression with an accuracy of 64.3% for mild and 73.8% for moderate NPDR, correctly detecting 100% of mild and up to 89% of moderate NPDR cases that progressed within 1 year [23].

A separate investigation developed a DL algorithm that predicted DR progression using a single baseline color fundus photograph (CFP). The model was evaluated for its ability to predict a two-step or greater worsening on the Early Treatment Diabetic Retinopathy Severity Scale (ETDRS DRSS) at 6, 12, and 24 months. It achieved AUC values of 0.68, 0.79, and 0.77, respectively, with the highest performance at 12 months. Interestingly, peripheral retinal fields (F3–F7) provided greater predictive value than central fields (F1–F2), and attribution maps confirmed the model’s focus on key microvascular abnormalities such as microaneurysms, hemorrhages, and exudate findings consistent with known markers of DR progression [24].

Another DL system was designed to predict the 2-year risk of DR development in patients without baseline retinopathy using color fundus photographs. It achieved AUC values of 0.79 with three-field images and 0.78 with one-field images in internal validation, and 0.70 in external validation using one-field images. Notably, this system outperformed traditional clinical risk factors like glycated hemoglobin (HbA1c). When combined with clinical data, performance improved further (AUC up to 0.81), enabling effective stratification into low-, medium-, and high-risk categories, with progression rates significantly higher in the high-risk group [25].

The DeepDR Plus system was developed to provide individualized risk assessment and time-to-progression prediction over a 5-year horizon using only fundus photographs. Trained on over 717,000 images and validated across internal and eight external multiethnic cohorts, the model demonstrated strong performance, with concordance indices ranging from 0.754 to 0.846 and integrating Brier scores between 0.153 and 0.241. It effectively stratified patients into high- and low-risk categories, accurately identifying progression to referable and vision-threatening DR with AUCs between 0.738 and 0.896. Importantly, integrating DeepDR Plus into clinical workflows in real-world settings safely extended screening intervals from 12 to approximately 32 months while maintaining a low delayed detection rate of just 0.18% [26].

A recent study from China aimed to enhance DR risk prediction by applying artificial intelligence to structured clinical data, addressing the limitations of conventional image-based screening and traditional statistical models. Utilizing 3000 patient records, 1500 with DR and 1500 without DR, from the Diabetes Complications Dataset, researchers compared five traditional machine-learning models, logistic regression, decision tree, naive Bayes, random forest, and support vector machine, with a deep neural network (DNN). The DNN consistently outperformed all traditional models across key metrics, achieving an accuracy of 0.778, F1 score of 0.776, and AUC of 0.833 compared to the next-best AUC of 0.831. SHAP analysis identified HbA1c, nephropathy, CHD, and diabetic peripheral polyneuropathy as key predictors, reinforcing the model’s clinical relevance and interpretability. The study acknowledged limitations, including the lack of DR severity stratification and the absence of external validation [27].

A study by Yang et al. demonstrated the utility of machine learning in predicting DR using clinical and biochemical data from 3000 diabetic patients, half diagnosed with DR. Eleven significant predictors, including HbA1c, glycated serum protein, 24 h urinary microalbumin, and urine protein creatinine ratio, were identified via multivariate logistic regression. Four models were developed: logistic regression, neural networks, random forest, and XGBoost. Tree-based models (random forest and XGBoost) outperformed others with accuracies of 95.67% and 94.67% and AUCs of 0.991 and 0.989, respectively. Feature importance analysis highlighted renal and glycemic markers, especially 24 h urinary microalbumin. While logistic regression was less accurate, it remains valuable for its interpretability. Limitations include the use of a single national database, lack of ensemble methods, and potential overfitting in complex models despite cross-validation, which may affect generalizability [28] (Table 2).

### 3.3. Limitations and Ethical Considerations

This literature review highlights key advancements and challenges in the use of AI for DR screening. However, several limitations were identified across the included studies that merit discussion. One key concern is the generalizability of AI algorithms. The data used to train these systems often lack diversity, which can lead to algorithmic bias and reduced accuracy when applied across different populations and geographic settings [1,7]. This limitation underscores the importance of incorporating more inclusive and representative datasets to avoid exacerbating existing health disparities.

Ethical and legal issues also remain insufficiently addressed. Although technical performance has been the primary focus of much research, critical aspects such as patient privacy, data ownership, and informed consent require more rigorous attention [4,29,30]. For example, the process of obtaining truly informed consent in AI-driven DR diagnostics is not well defined in current practice, particularly in primary care settings where time and understanding may be limited [31].

Additionally, variability among AI models presents challenges for clinical comparison and standardization. Differences in algorithm design, training methodologies, and evaluation criteria make it difficult to establish consistent benchmarks for safety, accuracy, and clinical efficacy [30,32]. This inconsistency can hinder regulatory approval and widespread adoption. Real-world integration of AI technologies into healthcare workflows is another area that requires further exploration. Several studies note that factors such as provider readiness, clinical workflow compatibility, and patient acceptance are often overlooked, despite being crucial for successful implementation [29,30,32]. Moreover, human-centered barriers, such as clinician skepticism or overreliance on AI decisions, highlight the need for comprehensive training and a balanced approach to clinical decision-making.

Finally, there is limited focus on the long-term impact and sustainability of AI in DR screening. Few studies evaluate the downstream effects on patient outcomes, healthcare costs, or systemic efficiency once these tools are implemented at scale [30]. Longitudinal research is needed to better understand how AI-driven solutions perform over time and under varying healthcare conditions.

## 4. Discussion

To evaluate the effectiveness of the various AI models applied in DR studies, we considered key performance metrics reported in each study, including sensitivity, specificity, accuracy, and area under the receiver operating characteristic curve. These quantitative indicators were crucial in comparing the diagnostic performance of different models. Additionally, we assessed practical outcomes such as accessibility in clinical settings, ease of implementation, and patient adherence to follow-up recommendations.

The application of AI in DR has evolved significantly over the past 2 decades. Earlier systems were largely rule-based, relying on manually designed features such as blood vessel segmentation, microaneurysm detection, and hard exudate identification [13,33]. These models exhibited limited scalability and lower diagnostic accuracy, with performance highly contingent on image quality and consistent feature extraction. With the emergence of deep learning, particularly convolutional neural networks, DR diagnostics have undergone a drastic shift. Systems like IDx-DR and EyeArt leverage large annotated datasets and hierarchical feature learning to automatically identify DR with high precision [34,35,36]. For instance, the IDx-DR system became the first FDA-approved autonomous AI for DR detection in 2018, achieving sensitivity and specificity values of 87.4% and 89.5%, respectively [34].

There has also been a shift in study design from early proof-of-concept models to large-scale randomized controlled trials (RCTs) and real-world validation studies. Earlier models were typically tested on small, homogeneous datasets, limiting generalizability [13,36]. In contrast, newer models have been validated across multiethnic and multinational cohorts, improving accuracy and supporting broader clinical deployment [35,37].

Beyond diagnosis, AI systems are increasingly being designed to predict disease progression, enabling more proactive and tailored patient management. Models integrating longitudinal imaging with clinical biomarkers (e.g., HbA1c levels, diabetes duration) have shown promise in forecasting DR severity and progression risk [38,39]. One anticipated application is the personalization of screening intervals. AI tools could stratify patients based on risk and recommend less frequent exams for low-risk individuals, conserving resources while maintaining safety [40]. This approach is currently being explored in real-world pilot programs. The integration of AI into telemedicine platforms is another promising direction, particularly in underserved or remote regions. Autonomous systems embedded in primary care or community clinics can screen patients and flag referable cases without requiring specialist input [41]. Such integration is already underway in several countries, enhancing accessibility and reducing delays in care [20].

A number of retinal imaging datasets have supported the development of AI models for diabetic retinopathy (DR) prediction and risk stratification. Among the most frequently used is the EyePACS dataset, derived from a U.S.-based screening program and widely known through the Kaggle DR Detection Challenge. It contains over 88,000 color fundus images from approximately 44,000 patients and has been pivotal for training deep-learning models due to its scale and grading annotations [22]. While these datasets differ in imaging protocols, patient demographics, and annotation detail, they collectively highlight the importance of standardized, diverse data sources in improving model generalizability and real-world applicability.

While this review highlights improvements in screening accuracy, accessibility, and follow-up adherence, broader implications on healthcare systems and clinical workflows warrant further attention. The integration of AI into electronic medical records (EMRs), AI–human diagnostic collaboration, and provider workload redistribution is a critical consideration, particularly with the FDA’s approval of autonomous systems such as IDx-DR [34]. Furthermore, concerns such as high false positive rates from instruments like EyeArt may increase unnecessary referrals and resource burden, raising cost-effectiveness concerns in already constrained health systems [42]. Legal and ethical considerations are equally vital. Guidance from organizations such as the World Health Organization (WHO) on ethics and governance of AI in health, as well as the European Union AI Act, provides critical ethical frameworks that emphasize human oversight, transparency, and accountability in AI-driven healthcare systems. These guidelines serve as essential policy anchors for developers and implementers to ensure AI tools align with principles of equity, safety, and public trust [41,43].

As AI continues to advance, its role in DR care is set to expand further, bridging gaps in healthcare access, enabling earlier intervention, and ultimately contributing to improved visual outcomes for patients around the world. Future research should focus on developing clinician–AI collaboration models, standardizing performance metrics, and exploring federated learning to improve data privacy and model generalizability. These steps are key to ensuring that AI tools for diabetic retinopathy are effectively integrated, ethically deployed, and equitable across diverse healthcare settings.

## 5. Conclusions

Artificial intelligence is emerging as a powerful tool in the diagnosis and management of DR, offering practical solutions to long-standing challenges such as delayed detection, limited access, and inconsistent follow-up. As the global burden of diabetes continues to rise, AI systems, particularly those based on deep learning, have demonstrated high diagnostic accuracy and the ability to identify referable and vision-threatening DR with impressive reliability. Just as importantly, they have shown potential to improve screening adherence and expand access to care, especially in underserved or resource-limited settings.

Findings from both randomized controlled trials and real-world studies highlight the impact of AI-supported screening programs in improving early detection and referral uptake across diverse healthcare environments. These tools are proving adaptable to different clinical workflows and populations, and ongoing developments in risk prediction and disease progression modeling suggest a growing role for AI in personalized, proactive care. Broader validation across diverse demographic and geographic populations, integration with health information systems, and the resolution of ethical concerns such as data privacy, transparency, and algorithmic bias are all critical to long-term success. Ensuring cost-effectiveness and sustainability, particularly in low-resource settings, will also be key to achieving widespread adoption.

As technology continues to mature, AI has the potential to shift diabetic retinopathy care toward earlier intervention, greater equity, and more individualized management. Realizing this potential will require continued collaboration among clinicians, researchers, policymakers, and communities working together to ensure that the benefits of AI are effective and accessible.

## Figures and Tables

**Table 1:** Summary of the reviewed literature on AI applications in screening programs and accessibility for DR.

Study	AI Application	Main Results/Conclusions
Mathenge et al., Ophthalmol Sci, 2022 [8]	AI-supported DR screening with immediate feedback	AI group had significantly higher referral adherence (51.5% vs. 39.6%, *p* = 0.048); faster referral presentation; age, sex, and rural residence influenced adherence.
Cleland et al., BMJ Open, 2024 [9]	AI-supported DR screening with SELENA + AI	Higher follow-up attendance, especially in low-resource settings; AI enabled immediate grading and counseling; included economic evaluation showing cost-effectiveness.
Wolf et al., Nat Commun, 2024 [10]	Autonomous AI screening in primary care	Exam completion: 100% (AI) vs. 22% (control); follow-up: 64% (AI) vs. 22% (control); multivariate analysis identified diabetes duration as the key predictor.
Noriega et al., JMIR Form Res, 2021 [11]	Automated deep learning analysis of fundus images	High accuracy (AUROC ~98%); improved ophthalmologist performance with AI assistance; mixed results with additional visual aids.
Dow et al., Clin Ophthalmol, 2023 [12]	AI-only vs. hybrid vs. human DR screening workflows	AI group had 69.2% follow-up (vs. ~12% others); faster result delivery (≤48 h) improved adherence; main barriers were patient-related.
Gardner et al., Br J Ophthalmol, 1996 [13]	Feature detection (vessels, exudates, hemorrhages) in fundus photos	High detection rates; DR detection: 88.4% sensitivity, 83.5% specificity; sensitivity could reach 99% with threshold adjustment.
Zhang et al., BMJ Open Diabetes Res Care, 2020 [14]	Deep learning-based DR screening across multiple sites	DR prevalence: 28.8%; referable DR: 24.4%; vision-threatening DR: 10.8%; good concordance with specialists (83%).
Heydon et al., Br J Ophthalmol, 2021 [15]	AI triage tool in English DESP	Sensitivity: 95.7% (100% for severe cases); specificity: 68–54%; high false-positive rate despite excellent detection of referable DR.
Lee et al., Diabetes Care, 2021 [16]	Comparison of seven AI algorithms vs. human graders	Variable sensitivity (50.98–85.90%); three algorithms matched/exceeded human graders; high NPV, moderate PPV; need for further validation.
He et al., Eye (Lond), 2020 [17]	AI vs. ophthalmologist grading in DR screening	High diagnostic performance: 91.18% sensitivity, 98.79% specificity for RDR; AI matched ophthalmologist detection rates.
Ming et al., Int Ophthalmol, 2021 [18]	AI grading vs. retina specialists	Good agreement (κ = 0.715); RDR: 84.6% sensitivity, 98% specificity; effective for DR/RDR detection in community clinics.
Bellemo et al., Lancet Digit Health, 2019 [19]	DL-based DR detection using ensemble model	AUC 0.973; RDR detection: 92.25% sensitivity, 89.04% specificity; excellent detection of vision-threatening DR and macular oedema.
Verbraak et al., Diabetic Medicine, 2019 [20]	IDx-DR 2.0 (autonomous detection DR in primary care)	Sensitivity 91%, specificity 84% (EURODIAB); high NPV; AUC 0.87–0.94; effective for reducing unnecessary referrals.
Pinto et al., Front Digit Health, 2025 [21]	NaIA-RD, a custom AI tool for DR screening integrated into GP workflow.	Increased GP sensitivity to 96.9%, strong agreement with ophthalmologists (Cohen’s kappa 0.818), reduced clinician workload by 4×, and matched or outperformed ophthalmologists across datasets.

Abbreviations: AI = Artificial Intelligence; DR = Diabetic Retinopathy; AUROC = Area Under the Receiver Operating Characteristic Curve; DESP = Diabetic Eye Screening Program; NPV = Negative Predictive Value; PPV = Positive Predictive Value; RDR = Referable Diabetic Retinopathy; DL = Deep Learning; AUC = Area Under Curve; EURODIAB = European Diabetes Study; GP = General Practitioner.

**Table 2:** Summary of the reviewed literature on AI applications in risk prediction and progression modeling for DR.

Study	AI Application	Main Results/Conclusions
Wang VY et al. Scientific Reports, 2024 [22]	Deep learning (ResNeXt) to classify DR severity and Mask-RCNN to detect microaneurysms using longitudinal fundus images	Combined model predicted progression from NRDR to RDR with F1-score of 0.422; AUC up to 0.971 for RDR detection; microaneurysm detection F1 score: 0.690
Silva PS et al. JAMA Ophthalmology, 2024 [23]	Automated machine learning using ultra-widefield retinal images for patients with mild/moderate NPDR	Accurately predicted 1-year DR progression: 100% detection in mild NPDR and 85–89% in moderate NPDR; AUPRC: 0.717 (mild), 0.863 (moderate); 3-year progression accuracy: 64.3–73.8%
Arcadu F et al. NPJ Digital Medicine, 2019 [24]	DL model to predict two-step or greater worsening on ETDRS scale from a single baseline fundus photo	AUCs: 0.68 (6 mo), 0.79 (12 mo), 0.77 (24 mo); peripheral fields (F3–F7) more predictive than central; model focused on microvascular abnormalities consistent with clinical markers
Bora A et al. The Lancet Digital Health, 2021 [25]	DL model predicting 2-year DR risk in patients without baseline DR using 1- or 3-field CFPs	Internal AUCs: 0.78–0.79; external AUC: 0.70; outperformed clinical factors (e.g., HbA1c); combining DL with clinical data increased AUC to 0.81; effective risk stratification
Dai L et al. Nature Medicine, 2024 [26]	DL system predicting 5-year individualized DR risk and time to progression using only fundus images	Trained on 717,000 images; concordance indices: 0.754–0.846; AUCs: 0.738–0.896; extended safe screening intervals from 12 to ~32 months; delayed detection rate: 0.18%
Gong et al. Frontiers in Medicine, 2025 [27]	Compared DNN vs. traditional ML models (logistic regression, decision tree, naive Bayes, random forest, SVM)	DNN achieved best performance (AUC: 0.833). SHAP analysis identified HbA1c, nephropathy, and CHD as top predictors
Yang et al. PLoS ONE, 2025 [28]	Compared logistic regression, neural networks, random forest, XGBoost	Tree-based models (random forest, XGBoost) had highest accuracy (~95%) and AUC (~0.99). Key predictors: renal and glycemic markers

Abbreviations: NRDR = Non-Referable Diabetic Retinopathy; RDR = Referable Diabetic Retinopathy; NPDR = Non-Proliferative Diabetic Retinopathy; AUC = Area Under Curve; F1 score = Harmonic Mean of Precision and Recall; Mask-RCNN = Mask Regional Convolutional Neural Network; AUPRC = Area Under the Precision-Recall Curve; ETDRS = Early Treatment Diabetic Retinopathy Study; CFP = Color Fundus Photograph; HbA1c = Glycated Hemoglobin; SHAP = SHapley Additive exPlanations.

## Abbreviations

The following abbreviations are used in this manuscript:

DR	Diabetic Retinopathy
AI	Artificial Intelligence
autoML	Automated Machine Learning
DESP	Diabetic Eye Screening Programme
NPDR	Non-Proliferative Diabetic Retinopathy
AUC	Area Under the Curve
AUROC	Area Under the Receiver Operating Characteristic Curve
CFP	Color Fundus Photograph
DL	Deep Learning
ETDRS	Early-Treatment Diabetic Retinopathy Study
F1-score	Harmonic Mean of Precision and Recall
GP	General Practitioner
HbA1c	Glycated Hemoglobin
Mask-RCNN	Mask Regional Convolutional Neural Network
NPV	Negative Predictive Value
NRDR	Non-Referable Diabetic Retinopathy
PPV	Positive Predictive Value
RDR	Referable Diabetic Retinopathy
AUPRC	Area Under the Precision-Recall Curve
EURODIAB	European Diabetes Study
